# iTRAQ-Based Quantitative Proteomic Analysis Reveals Proteomic Changes in Mycelium of *Pleurotus ostreatus* in Response to Heat Stress and Subsequent Recovery

**DOI:** 10.3389/fmicb.2018.02368

**Published:** 2018-10-09

**Authors:** Yajie Zou, Meijing Zhang, Jibin Qu, Jinxia Zhang

**Affiliations:** Institute of Agricultural Resources and Regional Planning, Chinese Academy of Agricultural Sciences, Beijing, China

**Keywords:** *Pleurotus ostreatus*, heat stress, TBARS, proteomics, recovery

## Abstract

High temperature is a key limiting factor for mycelium growth and development in *Pleurotus ostreatus*. Thermotolerance includes the direct response to heat stress and the ability to recover from heat stress. To better understand the mechanism of thermotolerance in *P. ostreatus*, we used morphological and physiological analysis combined with an iTRAQ-based proteomics analysis of *P. ostreatus* subjected to 40°C for 48 h followed by recovery at 25°C for 3 days. High temperature increased the concentrations of thiobarbituric acid reactive substances (TBARS) indicating that the mycelium of *P. ostreatus* were damaged by heat stress. However, these physiological changes rapidly returned to control levels during the subsequent recovery phase from heat stress. In comparison to unstressed controls, a total of 204 proteins were changed during heat stress and/or the recovery phase. Wherein, there were 47 proteins that responded to both stress and recovery conditions, whereas 84 and 73 proteins were responsive to only heat stress or recovery conditions, respectively. Furthermore, quantitative real-time PCR (qRT-PCR) confirmed differential expression of nine candidate genes revealed that some of the proteins, such as a mitogen-activated protein kinase (MAPK), phenylalanine ammonia-lyase (PAL), and heat shock protein (HSP), were also regulated by heat stress at the level of transcription. These differentially expressed proteins (DEPs) in mycelium of *P. ostreatus* under heat stress were from 13 biological processes. Moreover, protein–protein interaction analysis revealed that proteins involved in carbohydrate and energy metabolism, signal transduction, and proteins metabolism could be assigned to three heat stress response networks. On the basis of these findings, we proposed that effective regulatory protein expression related to MAPK-pathway, antioxidant enzymes, HSPs, and other stress response proteins, and glycolysis play important roles in enhancing *P. ostreatus* adaptation to and recovery from heat stress. Of note, this study provides useful information for understanding the thermotolerance mechanism for basidiomycetes.

## Introduction

*Pleurotus ostreatus*, also known as the oyster mushroom, is the third largest edible fungus produced in China. In 2015, the annual oyster mushroom production was estimated at 5.9 million tons, which represented 17% of the total edible fungi production for that year (data from China edible fungi association). *P. ostreatus* is highly valued for its superior texture, flavor, and nutritional quality as well as its demonstrated antioxidative, hypocholesterolemic, and antiatherogenic activities (Anandhi et al., 2013), antitumor properties (Jedinak and Sliva, 2008), and its ability to enhance the immune system (Jesenak et al., 2013). It is one of the most widely cultivated and consumed edible mushrooms in China due to its short growth time, high adaptability, and productivity.

High temperature stress or heat stress is defined as the temperature that when held beyond a critical threshold for a sufficient period of time will cause irreversible damage to growth and development. Heat stress for several days inhibits mycelium growth, impairs fruiting, and affects the quality of the mushroom (Chang and Miles, 2004). In China, *P. ostreatus* is usually cultivated within agricultural type facilities where it often encounters heat stress which reduces hyphae viability, delays fruiting, and leads to a decrease in production yield. Therefore, temperature is one of the crucial environmental factors that influence mushroom growth and development. Since tolerance to heat and other abiotic stressors is necessary for organisms to live in adverse environmental conditions and to function properly, the strategies of adaptation to high temperatures employed in *P. ostreatus* mycelium need further investigation. Previous studies exploring *P. ostreatus* response to high temperatures have only focused on physiological changes including cell programmed death (Song et al., 2014), cell membrane stability (Kong et al., 2012), mycelial micromorphology, and antioxidant systems (Meng et al., 2015), but few studies to date have investigated the changes in protein expression induced by heat stress during the thermotolerance response.

The present work aims to evaluate the quantitative changes in protein expression in the mycelium of *P. ostreatus* in response to heat stress using isobaric tags for relative and absolute quantitation (iTRAQ), an extremely powerful tool for identifying dynamic changes in proteomes on a global scale. Proteomic responses to abiotic stress have been widely studied in many plants and fungi including rice, wheat, barley, *Populus euphratica*, norway spruce, bitter gourd, grapevine (Liu et al., 2014), soybean (Das et al., 2016) *Flammulina velutipes* (Liu et al., 2017), *Agaricus bisporus* (Zhao-Ming et al., 2009), and *Boletus edulis* (Liang et al., 2007). iTRAQ has become a powerful method for investigating proteomic changes during various developmental stages (Hultinrosenberg et al., 2013). This technique has a high degree of sensitivity, and the lysine or N-terminal amine specific isobaric reagents of iTRAQ allow the identification and quantitation of multiple samples simultaneously.

In this study, iTRAQ labeling coupled with liquid chromatography-tandem mass spectrometry (LC-MS/MS) was used to identify differentially expressed proteins (DEPs) under heat stress and their subsequent recovery in order to better understand thermotolerance in mycelium of *P. ostreatus*. In addition, the morphological and physiological changes induced by heat stress were observed for each treatment. Moreover, we compared the changes at the proteomic and transcriptional levels under heat stress and their subsequent recovery conditions. These data might also provide new insights to the underlying molecular mechanisms of the proteins involved in thermotolerance in basidiomycetes.

## Materials and Methods

### Strain and Growth Conditions

*Pleurotus ostreatus* (CCMSSC 00389) was provided by the China Center for Mushroom Spawn Standards and Control. For all experiments, mycelia were grown in potato-dextrose agar (PDA) medium for 7 days at 28°C. Then 0.1 g of mycelia from solid medium were transferred to 100 mL of Difco^TM^ Potato Dextrose Broth medium in 250 mL erlenmeyer flasks. The mixture was dispersed using a liquid homogenizer, then returned to a culture flask, and incubated with shaking at 28°C and 160 rpm for 5 days.

### Heat and Recovery Treatments

The experimental plates included four different treatments: control treatment 1 (CK1): cultures were incubated with shaking at 28°C and 160 rpm for 5 days then held stationary at 28°C for 48 h. Heat stress (HS): cultures were incubated with shaking at 28°C and 160 rpm for 5 days then held stationary and subjected to heat stress at 40°C for 48 h. Recovery (RC): following the heat stress, cultures were incubated with shaking at 28°C and 160 rpm for 3 days. Control treatment 2 (CK2): cultures were incubated with shaking at 28°C and 160 rpm for 5 days then held stationary at 28°C for 48 h followed by incubation with shaking at 28°C and 160 rpm for 3 days.

### Measurement of Thiobarbituric Acid Reactive Substances (TBARS)

Thiobarbituric acid reactive substances (TBARS) were analyzed according to the method of Kong et al. (2012) with some modifications. The mycelia were ground into powder with liquid nitrogen, and then transferred into a 1.5 mL Eppendorf tube. Briefly, 0.5 mL of 5% TCA was added. Then the mixture was extracted for 10 min in ice water bath. The supernatants were collected by centrifuging at 10,000 × *g* for 10 min and mixed with 0.5 mL of 0.67% TBA in a new Eppendorf tube. The mixture was subsequently incubated at 95°C for 30 min, and then centrifuged at 10,000 × *g* for 10 min. The absorbance of the supernatant was measured at 532 and 600 nm wavelength using a UV-spectrophotometer (TU-1810, PERSEE, Beijing, China). All tests were performed in triplicate.

### Protein Extraction and iTRAQ Labeling

Protein extraction was performed according to a modified version of the trichloroacetic acid (TCA) acetone precipitation method described by Pratt et al. (2006) with some modifications. Triplicates of the frozen mycelia were combined equally for iTRAQ analysis. Approximately 500 mg of each ground up mycelia sample was combined with 10 mL of 10% m/v TCA in acetone and the samples were incubated at -20°C for 12 h. The samples were then centrifuged at 10,000 *g* for 15 min at 4°C. The supernatant was discarded without disturbing the pellets. The washing step with pre-cooled acetone was repeated three times until the pellets were white. The dried pellets were lyzed with 1 mL protein extraction reagent (4% SDS, 100 mM DTT, and 150 mM Tris-HCl, pH8.0). The pellets were dissolved by ultrasound (pulse on 10 s, pulse off 15 s, power 50 W) using 10 repeats and incubated at 100°C for 5 min. The solution was centrifuged at 40,000 *g* for 30 min at 4°C to remove insoluble impurities. The concentration of the protein was determined by the Brandford method using bovine serum albumin as a standard (Bradford, 1976), and the protein samples were analyzed by SDS-PAGE. For each sample, 200 μg protein were dissolved in 5 μL of 1 M dithiothreitol solution and incubated for 1 h at 37°C. Then, 20 μL of 1 M iodoacetamide solution was added and the samples were incubated for 1 h in darkness at room temperature. All samples were added to the filters and centrifuged at 12,000 *g* for 10 min. The collected liquid was discarded after centrifugation. Then, the filters were washed twice with 100 μL of UA buffer (8 M urea, 100 mM Tris-HCl, pH 8.0) and then three times with 100 μL of dissolution buffer (0.5 M triethylammonium bicarbonate at pH 8.5). The protein suspensions were digested with 40 μL of trypsin buffer (2 μg trypsin in 40 μL dissolution buffer) and incubated at 37°C for 12–16 h. After digestion with trypsin, the obtained peptides were dried by vacuum centrifugation and 100 μg of them were reconstituted in the dissolution buffer (0.5 M triethylammonium bicarbonate at pH 8.5) and processed according to the manufacturer’s protocol for iTRAQ Reagent Multi-Plex Kit (Applied Biosystems). Peptides from the digestion of the treatment samples CK1, CK2, HS, and RC were separately labeled using iTRAQ reagents with molecular masses of 114, 115, 116, and 117 Da. The pooled mixtures of iTRAQ-labeled peptides for each of the treatment groups were fractionated by strong cation exchange (SCX) chromatography.

### Liquid Chromatography-Tandem Mass Spectrometry (LC-MS/MS) and Data Analysis

Three replicates were run for the LC-MS/MS analysis. Digested peptide mixtures were pressure-loaded onto a fused silica capillary column packed with 3-μm dionex C18 material (RP; Phenomenex). The RP sections with 100 Å were 15 cm long and the column was washed with buffer A (water, 0.1% formic acid) and buffer B (acetonitrile, 0.1% formic acid). After desalting, a 5-mm, 300-μm C18 capture tip was placed in line with a quaternary HPLC (Agilent 1100) and analyzed using a 12-step separation.

The first step consisted of a 5-min gradient from 0 to 2% buffer B, followed by a 45-min gradient to 40% buffer B. Next, a 3-min gradient from 40 to 80% and 10-min 80% of buffer B was run followed by a 2-min buffer B gradient from 80 to 2%. Approximately 100 μg of tryptic peptide mixture was then loaded on to the columns and was separated at a flow rate of 0.5 μL/min using a linear gradient. As peptides were eluted from the micro-capillary column, they were electrosprayed directly into a micrOTOF-Q II mass spectrometer (BRUKER Scientific) with the application of a distal 180°C source temperature. The mass spectrometer was operated in the MS/MS (auto) mode. Survey MS scans were acquired in the TOF-Q II with the resolution set to a value of 20,000. Each survey scan (50–2,500) was followed by five data-dependent tandem mass (MS/MS) scans at 2HZ normalized scan speed.

Data were processed by ProteinPilot v.4.5 software (AB Sciex) and compared with the UniProt database. A 1.5-fold change cut off was used to categorize proteins as significantly changed. Proteins with iTRAQ ratios > 1.5 were considered to be up-regulated, and proteins with iTRAQ ratios < 0.67 were considered to be down-regulated. Information from the Gene Ontology (GO) was applied to the functional analysis. GO categories with a *P*-value < 0.05 were considered to be significant.

### Quantitative Real-Time PCR (qRT-PCR) Analysis

Total RNA was extracted from the mycelia using E.Z.N.A.TM Plant RNA Kit (Omega Bio-Tek) according to the manufacturer’s instructions. Briefly, 150 ng total cellular RNA was reverse transcribed using TIANScript RT Kit. The KAPA SYBR FAST qPCR Master Mix Kit (Kapa Biosystems, United States) and the ABI 7500 Real-Time PCR amplifier (Applied Biosystems, Foster City, CA, United States) were used for qPCR. All reactions were carried out in a total volume of 20 μL which contained 2 μL of diluted cDNA, 0.8 μL of primer mix (10 μM), 6.8 μL of nuclease-free water, 0.4 μL ROX Low, and 10 μL of SYBR Green mix. All reactions were performed in triplicate. The qPCR amplification procedures were as follows: 95°C for 3 min, 40 cycles of 95°C for 3 s, 60°C for 32 s, and a final extension at 72°C for 30 s. The GAPDH-encoding gene, *gapdh*, was used as the reference. Primers were designed using the DNAMAN software (**Table 2**) and were synthesized by Sangon Biotech Co., Ltd. (Shanghai, China).

### Bioinformatics Analyses

Functional classifications were performed using GO^1^, and pathway analysis was performed using KEGG^2^. The protein–protein interaction (PPI) network was analyzed using STRING (Search Tool for the Retrieval of Interacting Genes/Proteins) software^3^. The relative expression of the genes was calculated using the 2^-ΔΔ^*^C^*^t^ method (Livak and Schmittgen, 2001).

## Results

### Effect of Heat Stress Treatment and Subsequent Recovery on Morphological and Physiological Changes

**FIGURE 1:**
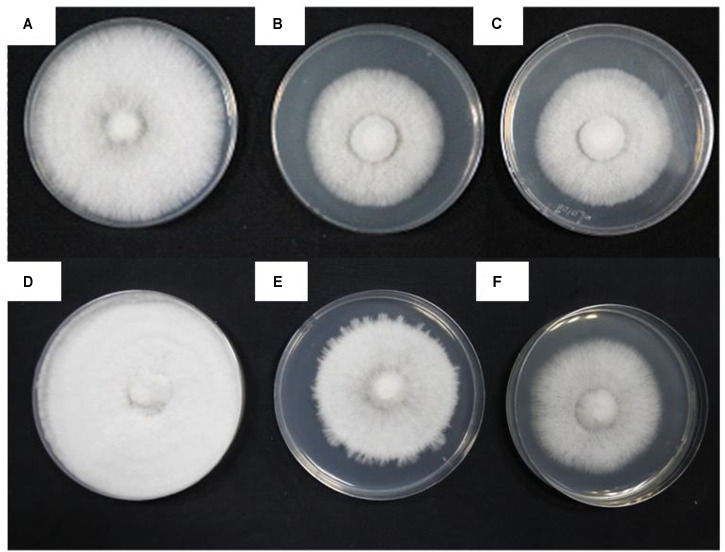
Different colony shapes and mycelial morphology of *P. ostreatus* in response to heat stress and recovery. **(A)** CK1; **(B)** incubated for 5 days at 28°C; **(C)** HS sample; **(D)** CK2; **(E)** RC sample; and **(F)** incubated for 3 days at 40°C following heat stress.

The four treatments were being incubated for 5 days at 28°C and then heat stress treatment for 48 h at 40°C (HS), 7 days at 28°C (CK1), 3 days at 28°C following the heat stress (RC), and 10 days at 28°C (CK2), respectively. The cultures for four treatments exhibited clearly different colony morphologies. Mycelia for CK1 produced vigorous aerial hyphae and the plate was almost fully colonized (**Figure 1A**), but the mycelium for HS treatment barely grew compared to the mycelium before heat stress (**Figures 1B,C**). Mycelia for CK2 grew thicker than that for CK1 and the plate was fully colonized (**Figure 1D**). Mycelia for RC treatment germinated vigorous aerial hyphae compared to that for following incubated at 40°C for 3 days again (**Figures 1E,F**). This result indicates that high temperature significantly inhibited the growth of mycelium.

**FIGURE 2:**
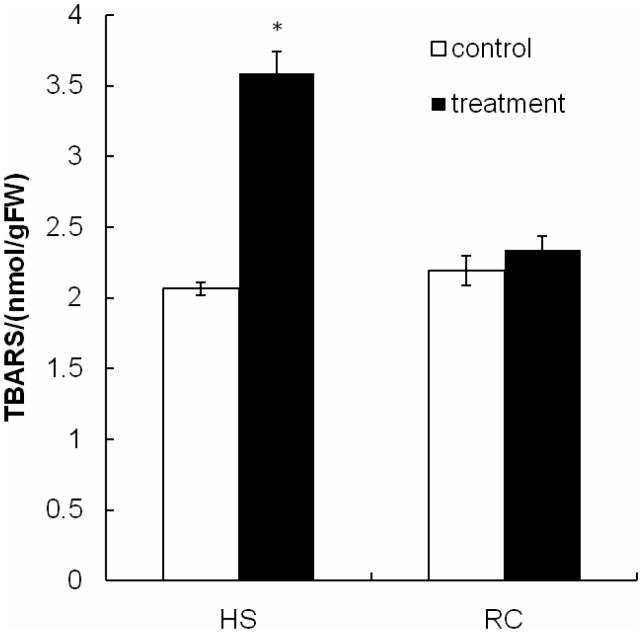
The TBARS concentration in mycelium after heat stress and recovery for *P. ostreatus*. HS group was cultivated for 5 days and subjected to heat stress for 2 days. RC group was allowed to recover for 3 days after exposure to heat stress. Data were analyzed by Duncan’s ANOVA test. Error bars represent the standard deviation of three replicates. The asterisks indicate the significance of differences between treatments and their corresponding controls (^∗^*P* < 0.05).

The present study investigated changes in cell membrane thermostability of *P. ostreatus* mycelium under heat stress and subsequent recovery. We used the TBARS concentration as an indicator of heat stress-induced peroxidation and destruction of lipid membranes (Kong et al., 2012). One-way ANOVA analysis showed that heat treatment (40°C for 48 h) significantly increased TBARS concentration in the mycelium compared with the CK 1 (**Figure 2**). TBARS content was as high as 3.586 nmol g^-1^ FW, 73.55% higher than that incubated at 28°C for 48 h (2.064 nmol g^-1^ FW). This result indicates that heat damages cell membranes by increasing the amount of reactive oxygen species (ROS) and that exposure to heat treatment for long periods of time may be lethal to the edible fungi mycelium. After subsequent recovery, there was no difference in TBARS concentration between RC (2.340 nmol g^-1^ FW) and control treatment (2.193 nmol g^-1^ FW; **Figure 2**), it is possible that the mycelia have a metabolic mechanism for repair of heat-induced cell membrane damage which allows a slow return to growth.

### Identification of Differentially Expressed Proteins in Response to Heat Stress and/or Recovery in *P. ostreatus* Mycelium as Revealed by iTRAQ Analysis

**FIGURE 3:**
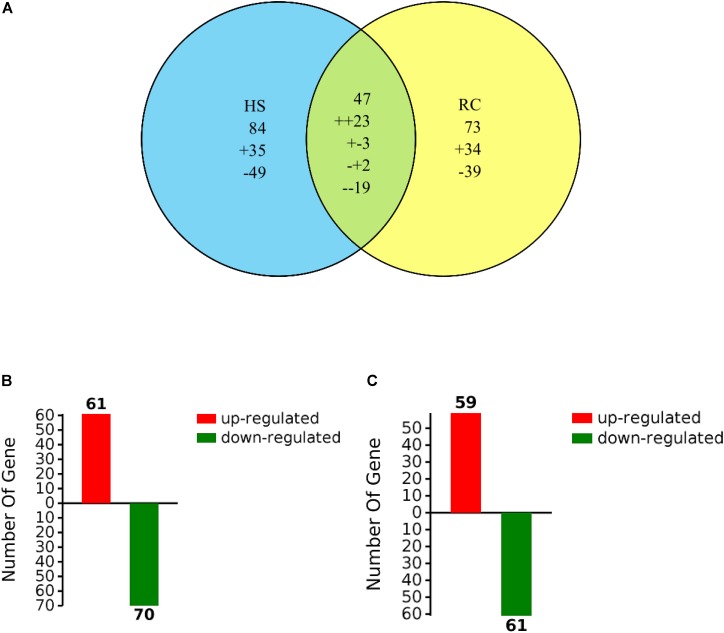
Venn diagram of differentially expressed proteins that were up- or down-regulated **(A)** by heat stress or recovery and total number **(B,C)** of identified DEPs from heat stress or recovery. The “+” and “-” indicate up- and down-regulated proteins, respectively. The “++” and “–” indicate up- and down-regulated under both heat stress and recovery, respectively. The “+-” indicates up-regulated under heat stress and down-regulated during recovery and the “-+” indicates down-regulated under heat stress but up-regulated during recovery.

Total proteins from three biological replicates were extracted from each of the four treatment groups of *P. ostreatus* (CK1, HS, CK2, and RC) and subjected to iTRAQ labeling and 2D LC-MS/MS analysis. Six hundred and eighty-six proteins were quantified with at least one significant peptide sequence and 204 of these characterized proteins were differentially expressed. Heat stress and recovery affected protein expression levels in various ways. Compared to the corresponding control levels, heat stress was associated with 61 proteins that were up-regulated and 70 that were down-regulated. In contrast, 59 were up-regulated and 61 were down-regulated after recovery (**Figure 3**). There were 84 (35 up- and 49 down-regulated) proteins and 73 (34 up- and 39 down-regulated) proteins responding to only heat stress or recovery, respectively, whereas 47 proteins were differentially expressed in both heat stress and recovery. Among these 47 proteins, 23 proteins were up-regulated under both heat stress and recovery and 19 proteins were down-regulated under both conditions. Three proteins were up-regulated under heat stress and down-regulated during recovery, while two proteins were down-regulated under heat stress but up-regulated during recovery (**Table 1**).

**Table 1:** Proteins with significant expression level changes in the mycelium under heat stress or subsequent recovery.

Uniprot ID	Proteins	Species	Percent coverage	No. of unique peptides	Fold change	
					
					HS/CK1	RC/CK2
**Developmental process**						
P17505	Malate dehydrogenase, mitochondrial	*Saccharomyces cerevisiae*	2.99	1	0.777	0.630
P29465	Chitin synthase 3	*Saccharomyces cerevisiae*	0.77	1	0.912	0.502
P53228	Transaldolase NQM1	*Saccharomyces cerevisiae*	3.6	1	0.931	0.520
P0CP66	Mitogen-activated protein kinase CPK1	*Cryptococcus neoformans*	7.1	1	1.010	1.780
Q12702	Protein phosphatase PP2A regulatory subunit B	*Schizosaccharomyces pombe*	4.75	2	1.298	2.146
O60041	Calmodulin	*Kluyveromyces lactis*	5.44	1	1.336	0.669
**Response to stimulus**						
Q99316	Protein disulfide isomerase MPD2	*Saccharomyces cerevisiae*	2.53	1	0.770	0.624
Q6CFX5	Serine/threonine-protein phosphatase 2A activator 1	*Yarrowia lipolytica*	2.08	1	0.827	0.599
Q12458	Putative reductase 1	*Saccharomyces cerevisiae*	2.56	1	0.750	1.947
**Organelle part**						
P28345	Malate synthase, glyoxysomal	*Neurospora crassa*	1.48	1	1.467	2.546
Q9P7Q8	Mo25-like protein	*Schizosaccharomyces pombe*	3.34	1	1.662	1.894
P0CR56	Pre-mrna-processing protein 45	*Cryptococcus neoformans*	2.19	1	0.633	0.621
Q9TEM3	2-Methylcitrate synthase, mitochondrial	*Emericella nidulans*	2.17	1	1.263	0.602
Q873W8	40S ribosomal protein S23	*Neosartorya fumigata*	7.59	1	0.763	2.320
O14036	Small nuclear ribonucleoprotein Sm D2	*Schizosaccharomyces pombe*	8.7	1	0.859	0.651
**Organelle**						
P15937	Acetyl-coa hydrolase	*Neurospora crassa*	1.9	1	1.001	0.524
P0CQ10	Cysteine protease ATG4	*Cryptococcus neoformans*	1.26	1	0.866	1.508
P18253	Peptidyl-prolyl cis-trans isomerase	*Schizosaccharomyces pombe*	4.94	1	1.567	1.608
Q4WHG1	Histone acetyltransferase esa1	*Neosartorya fumigata*	3.52	1	1.923	1.709
P58371	Subtilisin-like proteinase Spm1	*Magnaporthe oryzae*	4.66	1	0.582	0.552
P19848	Ubiquitin	*Coprinellus congregatus PE*	40.79	4	1.012	1.563
P33282	Uricase	*Emericella nidulans*	4.32	1	0.943	1.689
Q09923	Aldo-keto reductase yakc [NADP(+)]	*Schizosaccharomyces pombe*	5.88	2	0.855	0.604
B0DX25	Type 1 phosphatases regulator YPI1	*Laccaria bicolor*	5.73	1	1.467	0.541
**Catalytic activity**						
P43547	Putative aryl-alcohol dehydrogenase AAD6	*Saccharomyces cerevisiae*	3.77	1	1.639	0.583
O74187	Aldehyde dehydrogenase	*Agaricus bisporus* GN	4	1	1.058	1.696
P40108	Aldehyde dehydrogenase	*Davidiella tassiana* GN	2.02	1	0.932	0.628
Q6BSS8	Acyl-protein thioesterase 1	*Debaryomyces hansenii*	9.91	1	1.005	0.647
O94153	Imidazoleglycerol-phosphate dehydratase	*Phaffia rhodozyma* GN	6.83	1	1.091	0.642
Q92195	Phenylalanine ammonia-lyase (fragment)	*Agaricus bisporus* GN	5.63	1	5.107	1.525
P22011	Peptidyl-prolyl cis-trans isomerase	*Candida albicans*	4.94	1	1.213	0.597
Q03148	Pyridoxal 5’-phosphate synthase subunit SNZ1	*Saccharomyces cerevisiae*	2.69	1	0.987	0.492
C5PCB1	Subtilisin-like protease CPC735_066880	*Coccidioides posadasii*	2.02	1	0.035	0.301
D4AX50	Subtilisin-like protease 8	*Arthroderma benhamiae*	3.88	1	0.034	0.440
Q9HGY8	Triosephosphate isomerase	*Aspergillus oryzae*	9.56	2	0.665	0.590
A6YRN9	Trehalose phosphorylase	*Pleurotus pulmonarius*	30.72	20	0.859	0.652
P13228	Tryptophan synthase	*Neurospora crassa*	4.52	1	1.046	0.525
C5FZ57	Putative aspergillopepsin A-like aspartic endopeptidase MCYG_07979	*Arthroderma otae*	1.86	1	2.649	1.573
**Cell killing**						
P83467	Ostreolysin A6	*Pleurotus ostreatus*	16.67	2	0.710	0.348
**Cell**						
Q9P6S3	Up-regulated during septation protein 1	*Schizosaccharomyces pombe*	1.05	1	1.321	0.201
B0Y3B5	E3 ubiquitin ligase complex SCF subunit sconc	*Neosartorya fumigata*	4.43	1	0.439	0.623
**Molecular function regulator**						
Q7M4T5	Serine proteinase inhibitor IA-2	*Pleurotus ostreatus*	17.11	1	1.086	0.538
**Binding**						
Q4P2Q7	3-Hydroxyanthranilate 3,4-dioxygenase	*Ustilago maydis*	6.08	1	1.240	0.667
P78812	6-Phosphogluconate dehydrogenase, decarboxylating	*Schizosaccharomyces pombe*	4.67	2	0.912	0.611
Q9P7W3	Probable ATP-citrate synthase subunit 1	*Schizosaccharomyces pombe*	1.95	1	1.012	0.550
Q8NJN3	Acetyl-coenzyme A synthetase 2	*Candida albicans*	1.49	2	0.683	0.534
A8PDE3	Acetyl-coenzyme A synthetase	*Coprinopsis cinerea*	1.36	1	0.784	0.469
Q9Y702	Actin-1	*Schizophyllum commune*	52.53	4	1.000	0.656
P87072	Calcineurin subunit B	*Neurospora crassa*	5.75	1	0.938	1.664
Q2URJ3	F-actin-capping protein subunit beta	*Aspergillus oryzae*	3.76	1	1.033	0.592
Q96VB8	Peroxisomal catalase	*Candida boidinii*	3.57	2	0.655	0.367
Q01112	Cell division control protein 42 homolog	*Schizosaccharomyces pombe*	16.67	3	1.031	0.638
Q8SSJ5	Cell division control protein 48	*Encephalitozoon cuniculi*	3.46	1	0.619	0.602
Q06440	Coronin-like protein	*Saccharomyces cerevisiae*	1.23	1	0.690	0.359
Q4P804	COP9 signalosome complex subunit 5	*Ustilago maydis*	3.45	1	0.861	0.577
P09437	Cytochrome b2, mitochondrial	*Wickerhamomyces anomalus*	2.27	1	0.887	0.384
P0CQ75	ATP-dependent RNA helicase ded1	*Cryptococcus neoformans*	10.2	2	1.039	1.581
Q9P6U9	ATP-dependent RNA helicase ded1	*Neurospora crassa*	9.01	2	1.211	1.958
A7EJY3	ATP-dependent RNA helicase ded1	*Sclerotinia sclerotiorum*	10.91	3	2.706	1.694
Q2H5Z7	Translation machinery-associated protein 22	*Chaetomium globosum*	4.79	1	1.729	1.817
Q1E5R1	ATP-dependent RNA helicase DHH1	*Coccidioides immitis*	11.72	1	0.000	2.639
P0CY35	Elongation factor 1-alpha 1	*Candida albicans*	19	1	1.161	0.195
Q00251	Elongation factor 1-alpha	*Aureobasidium pullulans*	21.57	1	0.495	0.670
A5DPE3	Elongation factor 1-alpha	*Meyerozyma guilliermondii*	14.63	1	1.183	1.948
Q01765	Elongation factor 1-alpha	*Podospora curvicolla*	16.7	1	1.746	0.057
Q96X45	Elongation factor 2	*Neurospora crassa*	2.49	1	0.814	0.605
A8PZS4	Eukaryotic translation initiation factor 3 subunit F	*Malassezia globosa*	3	1	1.037	1.520
Q6BI20	Enolase 2	*Debaryomyces hansenii*	4.78	1	1.439	0.326
O74286	Enolase (fragment)	*Cunninghamella elegans PE*	6.65	1	0.976	0.639
P42894	Enolase	*Neocallimastix frontalis PE*	11.01	4	0.887	0.607
Q6W3C0	Enolase	*Tuber borchii*	6.59	1	1.030	0.663
O59948	Eukaryotic peptide chain release factor subunit 1	*Podospora anserina*	2.3	1	1.992	1.695
O74718	Eukaryotic peptide chain release factor GTP-binding subunit	*Schizosaccharomyces pombe*	2.27	1	1.334	2.007
P32604	Fructose-2,6-bisphosphatase	*Saccharomyces cerevisiae*	2.21	1	1.229	0.427
Q9Y804	Fanconi-associated nuclease 1 homolog	*Schizosaccharomyces pombe*	1.14	1	0.565	0.580
G8BAW7	Fatty acid synthase subunit alpha	*Candida parapsilosis*	0.48	1	2.979	1.879
P0CS61	Flap endonuclease 1	*Cryptococcus neoformans*	2.21	1	0.713	1.554
Q6BMK0	Glyceraldehyde-3-phosphate dehydrogenase	*Debaryomyces hansenii*	16.12	1	0.819	0.563
Q9UW96	Glyceraldehyde-3-phosphate dehydrogenase	*Pleurotus sajor-caju*	27.16	5	1.109	0.644
Q96UV5	Glutamine synthetase	*Hebeloma cylindrosporum*	11.86	1	0.736	0.644
Q8J1R3	Glutamine synthetase	*Suillus bovinus*	11.58	1	0.897	0.594
Q6C3E0	Glutamine synthetase	*Yarrowia lipolytica*	2.2	1	0.776	0.519
Q9C102	Putative glutamate synthase [NADPH]	*Schizosaccharomyces pombe*	1.94	1	0.412	0.662
Q6BZH1	78 kDa glucose-regulated protein homolog	*Debaryomyces hansenii*	4.1	1	1.591	0.361
Q4P6N0	ATP-dependent RNA helicase HAS1	*Ustilago maydis*	5.92	3	1.491	1.983
Q9P3U4	E3 ubiquitin-protein ligase dbl4	*Schizosaccharomyces pombe*	1.39	1	0.832	1.838
P40235	Casein kinase I homolog hhp1	*Schizosaccharomyces pombe*	9.04	3	0.878	1.578
O94586	Hit family protein 1	*Schizosaccharomyces pombe*	6.02	1	0.699	0.661
P0CP69	Mitogen-activated protein kinase HOG1	*Cryptococcus neoformans*	11.51	1	1.141	1.973
O74465	Helicase required for rnai-mediated heterochromatin assembly 1	*Schizosaccharomyces pombe*	1.2	1	0.798	0.583
P19882	Heat shock protein 60, mitochondrial	*Saccharomyces cerevisiae*	5.94	1	2.217	1.653
Q10265	Probable heat shock protein ssa1	*Schizosaccharomyces pombe*	7.3	1	1.148	0.548
P46587	Heat shock protein SSA2	*Candida albicans*	17.21	2	1.239	0.608
P18694	Heat shock 70 kDa protein 2	*Ustilago maydis*	16.12	1	1.135	0.601
Q8J2M3	Heat shock protein HSP82	*Ashbya gossypii*	3.41	3	2.482	2.027
P46598	Heat shock protein 90 homolog	*Candida albicans*	4.1	2	0.995	1.523
P31540	Heat shock protein hsp98	*Neurospora crassa*	2.16	2	4.707	1.752
Q4P331	ATP-dependent RNA helicase eif4a	*Ustilago maydis*	10.22	1	0.909	2.096
Q10475	Eukaryotic translation initiation factor 4 gamma	*Schizosaccharomyces pombe*	0.5	1	1.295	1.552
Q6BWA5	Inorganic pyrophosphatase	*Debaryomyces hansenii*	13.94	1	0.503	0.547
P0CO41	Jmjc domain-containing histone demethylation protein 1	*Cryptococcus neoformans*	0.91	1	0.555	1.632
A2QPN9	Adenylate kinase	*Aspergillus niger*	6.2	1	1.244	0.514
Q9P7I2	Calcium/calmodulin-dependent protein kinase type I	*Schizosaccharomyces pombe*	2.99	1	1.343	1.575
P48467	Kinesin heavy chain	*Neurospora crassa*	2.91	1	0.919	0.327
O94122	Pyruvate kinase	*Agaricus bisporus*	12.59	7	0.785	0.603
P55251	3-Isopropylmalate dehydratase	*Rhizomucor pusillus*	5.17	2	0.722	0.576
P49601	3-Isopropylmalate dehydratase	*Ustilago maydis*	4.14	1	0.679	0.664
Q9UUS2	Linoleate diol synthase	*Gaeumannomyces graminis*	0.94	1	1.041	1.939
Q10190	Large subunit gtpase 1	*Schizosaccharomyces pombe*	1.46	1	1.703	1.580
Q6FY67	ATP-dependent RNA helicase MAK5	*Candida glabrata*	1.09	1	1.034	1.601
Q00859	Mitogen-activated protein kinase	*Fusarium solani subsp*	10.42	2	0.640	0.636
Q4P460	Sulfate adenylyltransferase	*Ustilago maydis*	1.39	1	1.408	0.649
O14354	Mitochondrial genome maintenance protein mgm101	*Schizosaccharomyces pombe*	12.22	3	1.024	0.616
A5DEV6	DNA mismatch repair protein MSH3	*Meyerozyma guilliermondii*	1.04	1	1.118	2.631
B0CZ32	Methylthioribulose-1-phosphate dehydratase	*Laccaria bicolor*	6.33	1	1.112	0.309
A7TDZ8	Myosin-1	*Vanderwaltozyma polyspora*	2.14	1	0.777	1.763
P87115	UPF0202 protein C20G8.09c	*Schizosaccharomyces pombe*	1.55	1	1.375	1.650
Q5A599	Histidine protein kinase NIK1	*Candida albicans*	1.11	1	0.841	0.626
P53742	Nucleolar GTP-binding protein 2	*Saccharomyces cerevisiae*	4.73	2	1.476	1.840
O94268	25S rrna (cytosine-C(5))-methyltransferase nop2	*Schizosaccharomyces pombe*	1.48	1	1.556	1.820
O94514	Nucleolar protein 56	*Schizosaccharomyces pombe*	4.83	2	1.368	1.585
Q6BLA0	Phosphoglycerate kinase	*Debaryomyces hansenii*	8.41	1	0.683	0.228
Q6BJ75	Pre-rrna-processing protein PNO1	*Debaryomyces hansenii*	5.49	1	2.116	2.822
Q09792	Serine/threonine-protein kinase ppk8	*Schizosaccharomyces pombe*	1.36	1	0.737	1.559
O14126	26S protease regulatory subunit 6A	*Schizosaccharomyces pombe*	4.34	2	0.558	0.620
P31374	Serine/threonine-protein kinase PSK1	*Saccharomyces cerevisiae*	0.59	1	0.772	0.412
Q92462	E3 ubiquitin-protein ligase pub1	*Schizosaccharomyces pombe*	1.17	1	0.796	0.421
Q99148	Bifunctional purine biosynthetic protein ADE1	*Yarrowia lipolytica*	0.89	1	0.954	0.634
Q8X1T3	Pyruvate carboxylase	*Pichia angusta*	1.62	2	0.878	0.635
Q09794	Protein ura1	*Schizosaccharomyces pombe*	1.02	2	1.032	1.615
P38251	Replication factor C subunit 5	*Saccharomyces cerevisiae*	2.26	1	1.395	1.597
Q12196	Serine/threonine-protein kinase RIO1	*Saccharomyces cerevisiae*	1.45	1	1.592	1.508
P36602	Ribonucleoside-diphosphate reductase large chain	*Schizosaccharomyces pombe*	2.34	1	1.194	1.907
P21672	Ribonucleoside-diphosphate reductase large chain 2	*Saccharomyces cerevisiae*	1.84	1	1.817	2.059
P41805	60S ribosomal protein L10	*Saccharomyces cerevisiae*	3.62	1	1.401	2.012
Q758S7	60S ribosomal protein L11	*Ashbya gossypii*	12.07	2	1.139	1.506
O74895	60S ribosomal protein L15-A	*Schizosaccharomyces pombe*	3.48	1	1.100	1.541
P0CX23	60S ribosomal protein L20-A	*Saccharomyces cerevisiae*	4.65	1	0.992	1.545
P51997	60S ribosomal protein L25	*Puccinia graminis PE*	6.33	1	1.014	2.192
P0CX45	60S ribosomal protein L2-A	*Saccharomyces cerevisiae*	8.27	1	0.532	1.598
O60143	60S ribosomal protein L7-C	*Schizosaccharomyces pombe*	4.38	1	1.880	1.697
Q7SBD5	60S ribosomal protein L7	*Neurospora crassa*	6.85	2	1.045	1.605
O13672	60S ribosomal protein L8	*Schizosaccharomyces pombe*	5.02	1	1.167	1.575
Q03195	Translation initiation factor RLI1	*Saccharomyces cerevisiae*	1.64	1	0.865	1.563
O74633	DNA-directed RNA polymerase I subunit RPA2	*Neurospora crassa*	1.22	1	0.678	0.620
A5DCV3	DNA-directed RNA polymerase II subunit RPB1 (fragments)	*Meyerozyma guilliermondii*	0.95	1	0.583	0.477
Q4WEU2	DNA-directed RNA polymerase III subunit rpc3	*Neosartorya fumigata*	2.54	1	1.395	2.321
Q7S8R8	26S proteasome regulatory subunit rpn-1	*Neurospora crassa*	2.66	2	0.879	0.612
Q9P6N8	ATP-dependent rrna helicase rrp3	*Schizosaccharomyces pombe*	2.58	1	1.318	1.526
P0CT73	40S ribosomal protein S11-A	*Schizosaccharomyces pombe*	11.84	2	0.718	1.611
P06367	40S ribosomal protein S14-A	*Saccharomyces cerevisiae*	13.14	1	1.750	0.609
Q7SFJ9	40S ribosomal protein S16	*Neurospora crassa*	5.63	1	0.961	1.552
P0CT66	40S ribosomal protein S18-A	*Schizosaccharomyces pombe*	11.18	2	1.042	1.604
P0CT79	40S ribosomal protein S28-A	*Schizosaccharomyces pombe*	13.24	1	1.330	0.587
P52810	40S ribosomal protein S9	*Podospora anserina*	15.26	1	1.862	1.712
Q6BYK1	Pre-mrna-splicing factor RSE1	*Debaryomyces hansenii*	0.72	1	0.944	0.485
A8NYM5	U1 small nuclear ribonucleoprotein C	*Coprinopsis cinerea*	13.4	2	0.761	0.666
P17608	GTP-binding protein ryh1	*Schizosaccharomyces pombe*	9.45	1	1.449	1.724
A1CRG9	Small COPII coat gtpase sar1	*Aspergillus clavatus*	14.81	1	0.796	1.565
P0CR31	Small COPII coat gtpase SAR1	*Cryptococcus neoformans*	21.16	1	1.309	1.783
P32420	Succinate dehydrogenase [ubiquinone] iron-sulfur subunit, mitochondrial	*Ustilago maydis*	7.8	3	0.952	0.626
Q07953	Ribosome maturation protein SDO1	*Saccharomyces cerevisiae*	3.2	1	1.921	1.517
Q6FIY2	Guanine nucleotide-exchange factor SEC12	*Candida glabrata*	1.31	1	2.915	2.194
A8N5E5	Protein SEY1	*Coprinopsis cinerea*	1.91	2	1.259	1.572
Q6BHN9	Sorting nexin-41	*Debaryomyces hansenii*	1.04	1	0.849	0.493
P0CR63	Sorting nexin-4	*Cryptococcus neoformans*	1.62	1	1.073	1.608
Q6CWW9	Transcription elongation factor SPT5	*Kluyveromyces lactis*	0.87	1	0.599	0.346
Q4WHP3	Serine/threonine-protein kinase ste20	*Neosartorya fumigata*	1.72	1	1.348	1.505
Q4P5N0	Serine/threonine-protein kinase SMU1	*Ustilago maydis*	1.74	1	0.798	1.609
Q5AQL1	Alanine–trna ligase	*Emericella nidulans*	3.64	2	0.901	0.630
O43011	Histidine–trna ligase, mitochondrial	*Schizosaccharomyces pombe*	1.95	1	0.853	0.607
Q8SRH2	Probable threonine–trna ligase, cytoplasmic	*Encephalitozoon cuniculi*	1.25	1	1.733	1.991
O75005	Valine–trna ligase	*Schizosaccharomyces pombe*	1.12	1	1.058	1.681
P79008	Tubulin beta chain	*Coprinopsis cinerea*	43.82	4	0.384	0.607
P13393	TATA-box-binding protein	*Saccharomyces cerevisiae*	8.75	2	3.586	1.664
P78921	Probable T-complex protein 1 subunit theta	*Schizosaccharomyces pombe*	1.83	1	0.953	2.025
P41835	Thiamine biosynthetic bifunctional enzyme	*Saccharomyces cerevisiae*	1.85	1	0.714	1.575
P52495	Ubiquitin-activating enzyme E1 1	*Candida albicans*	0.98	1	1.061	0.576
O42939	Ubiquitin-activating enzyme E1-like	*Schizosaccharomyces pombe*	2.39	1	0.675	0.518
O13685	Ubiquitin-conjugating enzyme E2 13	*Schizosaccharomyces pombe*	6.76	1	0.879	0.590
O74196	Ubiquitin-conjugating enzyme E2-16 kda	*Colletotrichum gloeosporioides*	7.48	1	1.077	1.559
P31411	V-type proton atpase subunit B	*Schizosaccharomyces pombe*	6.16	1	0.899	0.321
A8NU66	Exportin-T	*Coprinopsis cinerea*	2.16	2	1.382	1.533
Q4P149	5’-3’ exoribonuclease 2	*Ustilago maydis*	1.3	1	1.051	0.383
O94432	Uncharacterized RNA-binding protein C660.15	*Schizosaccharomyces pombe*	3.38	1	0.895	0.534
O59731	Uncharacterized J domain-containing protein C3E7.11c	*Schizosaccharomyces pombe*	2.54	1	1.136	1.551
Q9P3U2	Uncharacterized AAA domain-containing protein C328.04	*Schizosaccharomyces pombe*	1.62	1	1.044	0.387
P53049	Oligomycin resistance ATP-dependent permease YOR1	*Saccharomyces cerevisiae*	0.61	1	0.522	0.538
**Reproductive process**						
Q9UTR7	Meiotic coiled-coil protein 3	*Schizosaccharomyces pombe*	1.26	1	1.345	1.970
**Signaling**						
P39958	Rab GDP-dissociation inhibitor	*Saccharomyces cerevisiae*	3.77	1	0.667	1.679
**Membrane part**						
Q99128	AP-1 complex subunit gamma-1	*Ustilago maydis*	1.03	1	0.950	1.547
O13349	ATP synthase subunit 4, mitochondrial	*Kluyveromyces lactis*	3.38	1	1.211	0.665
P39981	Vacuolar amino acid transporter 2	*Saccharomyces cerevisiae*	2.92	1	0.920	0.587
O13395	Chitin synthase 6	*Ustilago maydis*	1.69	1	0.888	1.593
P32074	Coatomer subunit gamma	*Saccharomyces cerevisiae*	0.86	1	0.966	1.544
Q01519	Cytochrome c oxidase subunit 6B	*Saccharomyces cerevisiae*	9.64	1	1.009	0.532
A1CJQ1	Probable dipeptidyl-aminopeptidase B	*Aspergillus clavatus*	0.98	1	0.325	0.505
Q7RVX9	Repressible high-affinity phosphate permease	*Neurospora crassa*	1.4	1	0.662	0.661
Q4I5R9	Peptidyl-prolyl cis-trans isomerase B	*Gibberella zeae*	5.65	1	1.310	1.819
Q7S7Z6	Peptidyl-prolyl cis-trans isomerase B	*Neurospora crassa*	4.56	1	0.992	0.583
Q4P2B6	Protein transport protein SEC31	*Ustilago maydis*	0.78	1	1.080	0.555
Q755G4	V-type proton atpase 16 kDa proteolipid subunit 2	*Ashbya gossypii*	10.98	1	1.744	1.662
B0E2U2	Vacuolar protein sorting/targeting protein 10	*Laccaria bicolor*	0.75	1	1.002	0.638
O13941	Uncharacterized beta-glucan synthesis-associated protein C23H3.11c	*Schizosaccharomyces pombe*	1.43	1	0.200	0.524


### Functional Categorization Analysis

**FIGURE 4:**
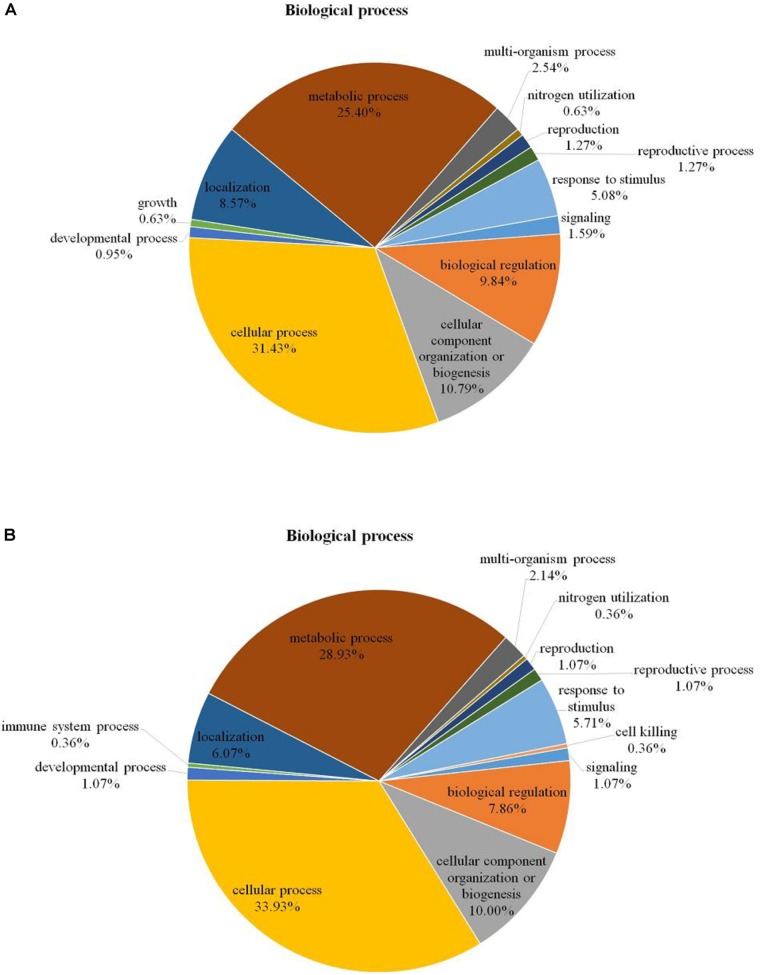
Bioinformatics analysis of DEPs responsive to heat stress **(A)** and subsequent recovery **(B)** in *P. ostreatus* mycelia compared to the control group through gene ontology (GO) in biological process (BP).

**FIGURE 5:**
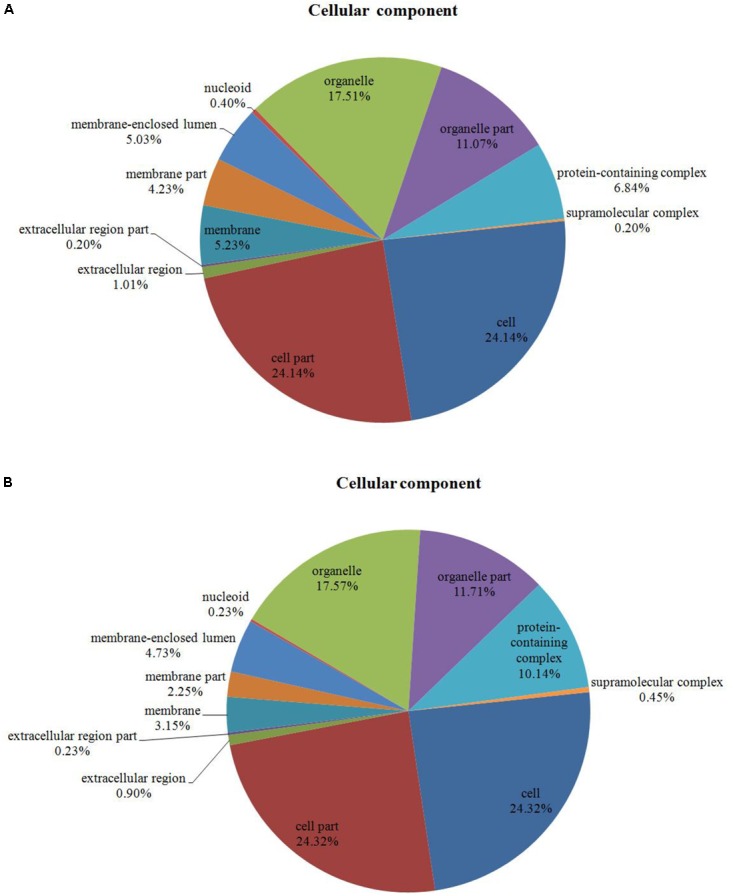
Bioinformatics analysis of DEPs responsive to heat stress **(A)** and subsequent recovery **(B)** in *P. ostreatus* mycelia compared to the control group through gene ontology (GO) in cell component (CC).

**FIGURE 6:**
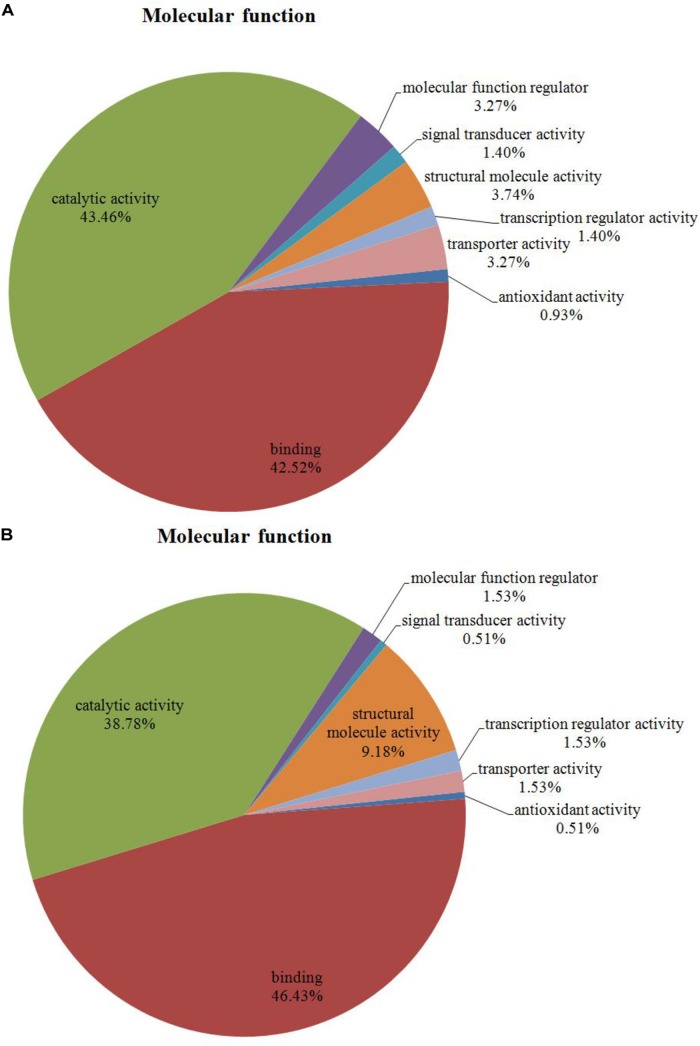
Bioinformatics analysis of DEPs responsive to heat stress **(A)** and subsequent recovery **(B)** in *P. ostreatus* mycelia compared to the control group through gene ontology (GO) in molecular function (MF).

Among the 204 DEPs, eight were characterized as hypothetical or unknown proteins using *P. ostreatus* genomics information published in uniprot^4^. To gain functional information about these proteins, BLASTP^5^ was used to search for homologous proteins against the NCBI non-redundant protein database. GO annotations enrichment, which was classified into biological process, cell components, and molecular function. The results showed that the DEPs identified in the mycelium under heat stress and recovery were primarily involved in cellular, metabolic, multi-organism, reproductive, and developmental processes; biological regulation; localization; nitrogen utilization; cellular component organization or biogenesis; reproduction; response to stimulus; signaling biological processes, whereas growth biological processes detected in HS (**Figure 4A**), and cell killing and immune system process detected in RC (**Figure 4B**). With regard to the cellular components, most DEPs were associated with organelle, organelle part, protein-containing complex, supramolecular complex, cell, cell part, nucleiod, membrane-enclosed lumen, membrane part, membrane, extracellular region part, extracellular region, but the proportions of molecular function are different in each treatment (**Figure 5**). Under the category of molecular function, most DEPs in the mycelium under heat stress and recovery were correlated with catalytic activity; binding; molecular function regulator; signal transducer activity; structural molecule activity; transcription regulator activity; transporter activity; antioxidant activity, but the proportions of molecular function are different in each treatment (**Figure 6**).

**FIGURE 7:**
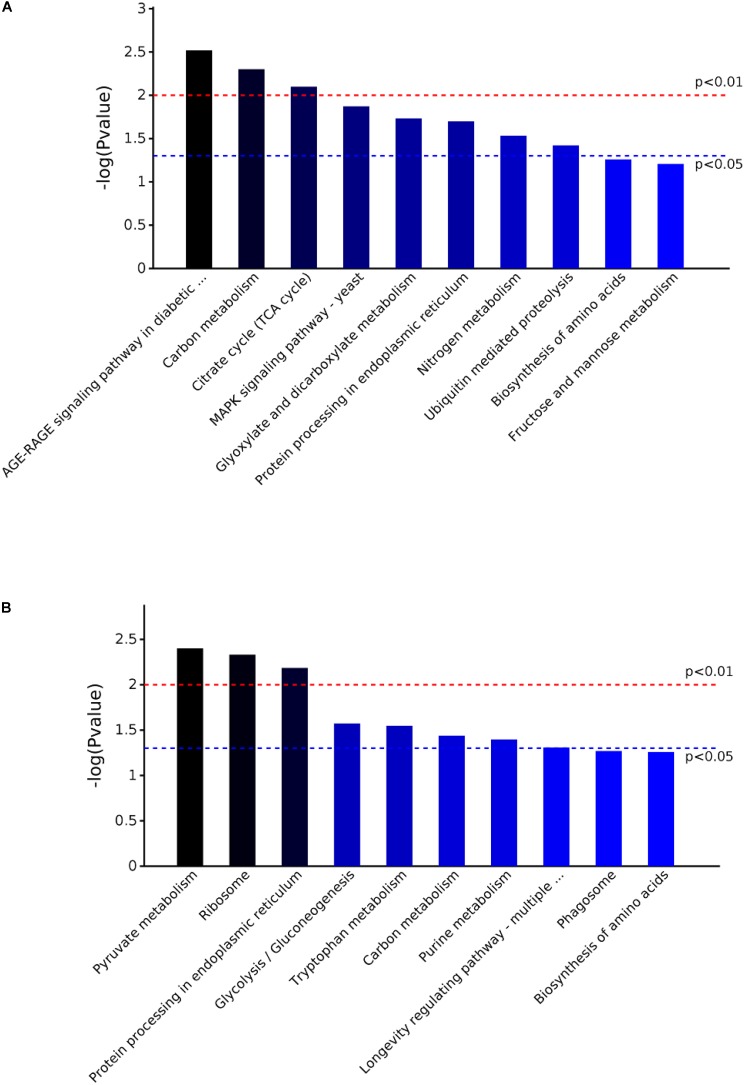
The KEGG pathway enrichment analysis of the DEPs in *P. ostreatus* mycelium under heat stress **(A)** and subsequent recovery **(B)**. Top 10 enrichment in KEGG pathway maps of the DEPs. *P*-value was calculated using Fisher’s exact test.

The KEGG pathway and enrichment analysis indicated that the DEPs in the mycelium under heat stress were highly enriched in AGE-RAGE signaling pathway in diabetic complications; carbon metabolism; citrate cycle (TCA cycle); MAPK signaling pathway; glyoxylate and dicarboxylate metabolism; protein processing in endoplasmic reticulum; nitrogen metabolism; ubiquitin mediated proteolysis; biosynthesis of amino acids; fructose and mannose metabolism (**Figure 7A**). While the DEPs in the mycelium under recovery were highly enriched in pyrvate metabolism; ribosome; protein processing in endoplasmic reticulum; glycolysis/gluconeogenesis; tryptophan metabolism; purine metabolism; longevity regulating pathway; phagosome; and biosynthesis of amino acids (**Figure 7B**).

### String Analysis of Protein–Protein Interactions for DEPs

**FIGURE 8:**
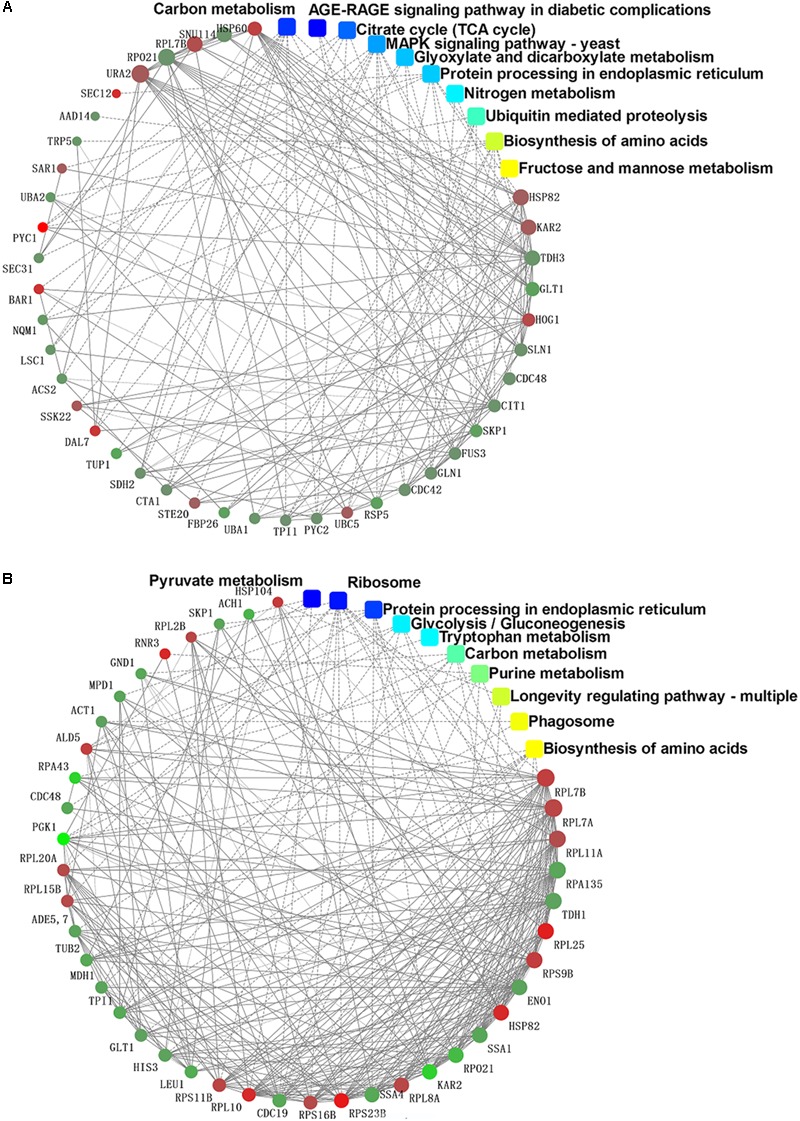
Protein–protein interaction network analysis among the significantly expressed proteins in *P. ostreatus* mycelium under heat stress **(A)** and subsequent recovery **(B)** using String software.

The PPIs whose combined score was >0.9 were used to build network using Cytoscape tool in each group. It was of note that the DEPs in the mycelium under heat stress of top 10 enrichment in KEGG pathway formed three subsets of protein interaction networks: carbohydrate and energy metabolism, signal transduction, and proteins metabolism (**Figure 8A**), while in the mycelium under recovery of top 10 enrichment in KEGG pathway formed differently compared to HS (**Figure 8B**). This indicated that proteins in this network played important functions in redox homeostasis, response to stress, signal transduction, and protein metabolism.

### Transcriptional Expression Analysis of Selected Proteins as Revealed by qRT-PCR

**FIGURE 9:**
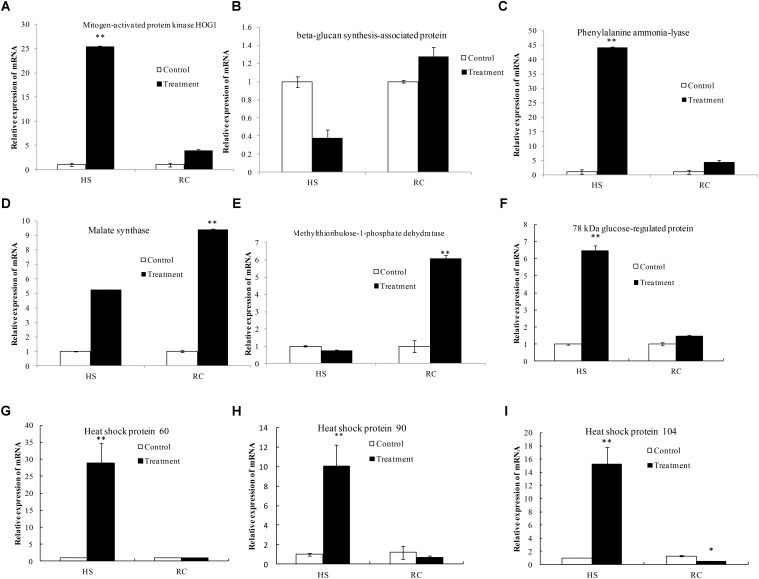
Transcriptional expression analysis of representative proteins as revealed by qRT-PCR. The relative mRNA expression levels of matched differentially abundant proteins including mitogen-activated protein kinase HOG1 **(A)**, beta-glucan synthesis-associated protein **(B)**, phenylalanine ammonia-lyase **(C)**, malate synthase **(D)**, methylthioribulose-1-phosphate dehydratase **(E)**, 78 kDa glucose-regulated protein homolog **(F)**, heat shock protein 60 **(G)**, heat shock protein 90 **(H)**, and heat shock protein 104 **(I)**. *Gapdh* was used as the reference gene. Mean values and standard deviations of three biological replicates are shown. The asterisks indicate the significance of differences between treatments and their corresponding controls (^∗∗^*P* < 0.01, ^∗^*P* < 0.05).

The data used in this study were subjected to rigorous statistical and bioinformatics analysis to eliminate possible errors as by Liu et al. (2017). To provide further information of the correspondence between proteins and their mRNA expression patterns, quantitative real-time PCR (qRT-PCR) was performed to investigate the dynamic transcriptional expression patterns of nine representative DEPs. The summarized primer data of nine representative DEPs are shown in **Table 2**. After heat treatment and recovery, the changes of the mRNA levels in eight genes correlated with changes at the protein levels as indicated by iTRAQ analysis, this included a *mapk*HOG1, β-*gs, pal, m*-1-*pd, hsp60, grp78, hsp90*, and *hsp104* (**Figures 9A–C, E–I**). The expression of the genes agreed with proteomics results (**Table 1**). The mRNA of *ms* showed a up-regulated trend in the mycelium under recovery (**Figure 9D**); however, *ms* had a lower protein expression level (**Table 1**). The expression of *ms* genes was not in accordance with proteomics due to translational or post-translational regulation. The result is generally consistent with those of a previous report (Vedeler et al., 2012; Liu et al., 2017).

**Table 2:** Primer sequences used for reverse transcription PCR.

Gene	Primer name	Primer sequence (5′-3′)
*hsp60*	*hsp60*-F	CAAGGACTGTGGCTGTT
	*hsp60*-R	TTTCTCTCAAGGATAAG
*grp78*	*grp78*-F	AGGCTGTCGCTTATGGTG
	*grp78*-R	AAGACGGTAGGCTGGTTGT
*hsp90*	*hsp90*F	TTACCAACGACTGGGAGGA
	*hsp90*R	GAAGACACGGCGGACATA
*hsp104*	*hsp104*-F	TCTGCGATGGCTTCTGGG
	*hsp104*-R	GGCGGAAGATGGACGAAC
*gapdh*	*gapdh*-F	ACCTTGAGACTTACGACCCG
	*gapdh*-R	TGTTGTTGACACTGCGACCT
*pal*	*pal*-F	ACGGAGGAAGAGGAGATG
	*pal*-R	ATGAACAAGCGAACAGGAT
*gs*	*gs*-F	GTCGGATAGAGATAGCAAGTAT
	*gs* -R	GTGGTTCAAGTTCGTCAGA
*mpd*	*mpd*-F	ATACTCAGATGTGCCAGAC
	*mpd*-R	GTAGACAGCGAACAGGAA
*mapk*	*mapk*-F	ATACTCAGATGTGCCAGAC
	*mapk*-R	GTAGACAGCGAACAGGAA
*ms*	*ms*-F	CATCACTGTCGCCTATGTC
	*ms*-R	GTCGCTGGTCAAGAACTC


## Discussion

One of the many locations for heat stress injury in cell is the membrane. TBARS is the product of lipid peroxidation in fungi. With the increase of temperature, the levels of membrane lipid peroxidation will be increased (Kong et al., 2012). In this study, we investigated the morphological and TBARS content of the mycelium in *P. ostreatus* under heat stress and subsequent recovery (**Figures 1, 2**). These results showed that the mycelium of *P. ostreatus* were damaged under heat stress at 40°C for 48 h, but they subsequently recovered at 25°C for 3 days. These results indicated that *P. ostreatus* mycelia suffered greater damage on membrane lipid after high temperature (40°C) and *P. ostreatus* mycelia treated with 40°C for 48 h was a suitable treatment for studying changes in extracellular metabolites.

In this study, taking advantage of iTRAQ-based quantitative proteomics technology, we investigated the response of *P. ostreatus* to heat stress and recovery on a proteome-scale. More than 204 proteins, which were almost 29.73% of all detected 686 proteins, were up- or down-regulated in heat-treated and recovery in *P. ostreatus*, indicating that heat strongly influences fungi physiology. The biological relevance of these DEPs in the *P. ostreatus* under heat stress and subsequent recovery are discussed below.

### Carbohydrate and Energy Metabolism

Heat stress alters the abundance of many proteins involved in carbohydrate and energy metabolism, which was mainly included the citrate cycle (TCA cycle), glycolysis, glyoxylate and dicarboxylate metabolism, and nitrogen metabolism in *P. ostreatus* mycelia. The TCA cycle is an important aerobic pathway involved in the conversion of carbohydrates, fats, and proteins to form energy (Cetica et al., 2003), which starts with acetyl-CoA, the activated form of acetate, derived from glycolysis and pyruvate oxidation for carbohydrates and from beta oxidation of fatty acids, and it is noteworthy that four proteins involved in the TCA process, including 2-methylcitrate synthase, succinate dehydrogenase, ATP-citrate synthase, and pyruvate dehydrogenase had lower expression levels in mycelia after heat stress but recovered to control levels after subsequent recovery. Pyruvate dehydrogenase is an enzyme component of the multienzyme pyruvate dehydrogenase complex and is involved in the formation of cellular energy during the TCA cycle. 2-Methylcitrate synthase catalyzes the synthesis of (2S,3S)-2-methylcitrate from propionyl-CoA and oxaloacetate and also from acetyl-CoA. In this study, the abundance of Pyruvate dehydrogenase and 2-methylcitrate synthase decreased under heat stress. This suggests that the TCA cycle was inhibited in *P. ostreatus* after 48 h of heat stress treatment (Rice and Bayles, 2008). As shown in **Table 3**, there are complex protein abundance change patterns in acute normal culture to heat stress transfer in mycelia of *P. ostreatus* at the molecular level. There were six kinds of enzymes involved in glycolysis that showed no significant change in expression under heat stress, but were down-regulated after subsequent recovery; these included glyceraldehyde-3-phosphate dehydrogenase, phosphoglycerate kinase, pyruvate kinase, and enolase. Overall, the results indicate that the glycolytic pathway was not affected by heat stress and that the TCA process was suppressed by the heat stress despite the return to control levels during recovery. These results suggest that the glycolytic pathway is more heat-resistant than the TCA cycle in the respiration of mycelium of *P. ostreatus* during heat stress.

**Table 3:** Variation of proteins involved in respiration under heat stress and subsequent recovery.

Uniprot ID	Mascot score	Fold change		Species	Description
				
		HS/CK1	RC/CK2		
Q9UW96	1802.37	1.109	0.644	*Pleurotus sajor-caju*	Glyceraldehyde-3-phosphate dehydrogenase
O94122	496.25	0.785	0.603	*Agaricus bisporus*	Pyruvate kinase
Q6BLA0	451.99	0.683	0.228	*Debaryomyces hansenii*	Phosphoglycerate kinase
Q9HGY8	271.03	0.665	0.590	*Aspergillus oryzae*	Triosephosphate isomerase
P17505	168.90	0.777	0.630	*Saccharomyces cerevisiae*	Malate dehydrogenase
P42894	101.22	0.887	0.607	*Neocallimastix frontalis*	Enolase-EMP
A8PDE3	90.58	0.469	1.253	*Coprinopsis cinerea*	Acetyl-coenzyme A synthetase
Q6BMK0	69.96	0.563	0.701	*Debaryomyces hansenii*	Glyceraldehyde-3-phosphate dehydrogenase
Q8NJN3	63	0.534	1.325	*Candida albicans*	Acetyl-coenzyme A synthetase 2
P78812	41.12	0.912	0.611	*Schizosaccharomyces pombe*	6-Phosphogluconate dehydrogenase
P32420	37.21	0.626	0.715	*Ustilago maydis*	Succinate dehydrogenase
Q8X1T3	33.30	0.635	0.767	*Pichia angusta*	Pyruvate carboxylase
O74286	NA^∗^	0.976	0.639	*Cunninghamella elegans*	Enolase
Q6W3C0	NA^∗^	1.030	0.663	*Tuber borchii*	Enolase
Q6BI20	NA^∗^	1.439	0.326	*Debaryomyces hansenii*	Enolase 2
Q9P7W3	NA^∗^	0.550	0.944	*Schizosaccharomyces pombe*	Probable ATP-citrate synthase


*NA^∗^, the proteins were not quantified under heat stress or subsequent recovery.*

### Signal Transduction

Reactive oxygen species are found in normal living organisms where they are constantly being produced under the oxidative stress caused by toxic heavy metals, heat shock, inflammation, ionizing irradiation, immune responses, and environmental stimuli (Zhai et al., 2018). Studies have shown that antioxidant enzymes can remove and reduce ROS produced by metabolic stress conditions in an attempt to maintain homeostatic equilibrium. As shown in **Table 4**, 18 dysregulated proteins involved in the heat stress response were detected. Four of the key proteins involved in the redox reactions, i.e., peroxisomal catalase, thiamine biosynthetic bifunctional enzyme, linoleate diol synthase (LDS), and uricase which play a role in protecting against oxidative stress resulted up-regulated during heat stress. For example, expression of LDS is increased by 1.94-fold under heat stress, which converted oleic acid, linoleic acid, and α-linolenic acid to 7,8-dihydroxy fatty acids, but this enzyme showed no activity when γ-linolenic acid, eicosatrienoic acid, arachidonic acid, and eicosapentaenoic acid were used as substrates (Brodowskys et al., 1992). Catalase, universal in many fungi, rapidly catalyzes the decomposition of hydrogen peroxide into less-reactive gaseous oxygen and water molecules protecting the cell from oxidative damage due to accumulation of ROS (Isobe et al., 2006). In our study, the expression of CAT was not significantly changed under heat stress; however, the expression was significantly lower after recovery. Similar results were observed for Po-cat2 activity under heat stress which may be caused by the inhibition of the overall protein synthesis under stressful conditions or by alternative H_2_O_2_ detoxification pathways function (Wang et al., 2017). CAT and ascorbate peroxidase (APX), another key detoxifying enzyme, act together to alleviate the aggregation of H_2_O_2_ and other ROS resulting from uric acid oxidation catalyzed by uricase. Uricase is increased 1.7-fold under heat stress. In addition, another redox enzyme, thiamine biosynthetic bifunctional enzyme, is increased 1.6-fold under heat stress (**Table 3**). It is clear that these key enzymes participate in the removal of ROS and protecting the cells from oxidation damage (Sun et al., 2013).

**Table 4:** Variation of proteins involved in abiotic stress and redox under heat stress and/or subsequent recovery.

Uniprot ID	Mascot score	Fold change		Species	Description
				
		HS/CK1	RC/CK2		
P18694	2423.70	1.135	0.601	*Ustilago maydis*	Heat shock 70 kDa protein 2
P46587	2197.33	1.239	0.608	*Candida albicans*	Heat shock protein SSA2
Q10265	2022.53	1.148	0.548	*Schizosaccharomyces pombe*	Probable heat shock protein ssa1
P46598	476.98	1.523	0.982	*Candida albicans*	Heat shock protein 90 homolog
Q96VB8	326.56	0.655	0.367	*Candida boidinii*	Peroxisomal catalase
Q6BZH1	239.05	1.591	0.361	*Debaryomyces hansenii*	78 kDa glucose-regulated protein homolog
Q8J2M3	186.14	2.482	2.027	*Ashbya gossypii*	Heat shock protein HSP82
P31540	136.87	4.707	1.752	*Neurospora crassa*	Heat shock protein Hsp98
Q9UUS2	40.64	1.939	0.789	*Gaeumannomyces graminis var. graminis*	Linoleate diol synthase
P19882	95.49	2.217	1.653	*Saccharomyces cerevisiae*	Heat shock protein 60
P0CP69	89.45	1.973	1.116	*Cryptococcus neoformans var. neoformans*	Mitogen-activated protein kinase HOG1
Q00859	79.36	0.636	1.243	*Fusarium solani*	Mitogen-activated protein kinase
Q5A599	43.21	0.626	0.974	*Candida albicans*	Histidine protein kinase
P33282	38.49	1.689	1.332	*Emericella nidulans*	Uricase
P41835	37.74	1.575	1.398	*Saccharomyces cerevisiae*	Thiamine biosynthetic bifunctional enzyme
O59731	35.21	1.551	0.945	*Schizosaccharomyces pombe*	Uncharacterized J domain-containing protein
Q09792	NA^∗^	1.559	1.349	*Schizosaccharomyces pombe*	Serine/threonine-protein kinase ppk8
Q4WHP3	NA^∗^	1.505	1.087	*Neosartorya fumigata*	Serine/threonine-protein kinase ste20


*NA^∗^, the proteins were not quantified under heat stress or subsequent recovery.*

Most of the proteins involved in oxidative stress are heat shock proteins (HSPs) with chaperone activity that belong to five conserved classes, HSP60, HSP70, HSP90, HSP100, and the small heat shock proteins (sSHPs). In fungi as well as most eukaryotic cells, HSPs are involved in various routine biological processes such as transcription, translation and post-translational modifications, protein folding, and aggregation and disaggregation of proteins (Tiwari et al., 2015). In our experiments, the expression of Hsp60 increased 2.2-fold under heat stress. This result agrees with results from *Paracoccidioides brasiliensis* which showed that Hsp60 is also up-regulated in response to thermal stress (Felipe et al., 2005). This might suggest that Hsp60 may have important functions in alleviating heat stress in *P. ostreatus* mycelium. The *P. brasiliensis* study also identified additional heat shock proteins which are essential for cell viability: Hsp70-2, 70-kDa HSPs of the SSA subfamily, Hsp70/SSA1 and Hsp70/SSA2, as well as glucose-regulated protein 78 kDa (GRP78). The Hsp70 protein family both under normal or environmental conditions of stress prevent protein aggregation and promote protein folding (Frydman, 2001). In addition, they participate in protein input and transfer processes and promote the degradation of unstable proteins. Moreover, Hsp70 has been reported to accumulate during the heat stress response in several organisms (Sørensen et al., 2003; Lee et al., 2007), and the expression of GRP78, a member of the Hsp70 family, increased by 1.6-fold under heat stress and then decreased to 0.36-fold after recovery. Interestingly, it has been shown that GRP78 promotes endoplasmic reticulum protein complex assembly^6^. Two Hsp90 family proteins, Hsp82 and Hsp90 homolog, were also evaluated during heat stress and recovery. Hsp82 expression increased 2.5-fold under heat stress and then decreased to twofold after recovery. In contrast, the Hsp90 homolog was not affected by thermal stress. Members of the Hsp90 family are molecular chaperones that mediate the folding of a defined set of signaling proteins involved in repair, signal transduction, cell-cycle regulation, protein degradation, and transport (Richter and Buchner, 2001; Pratt et al., 2006). Studies have shown that when *P. euphratica* was subjected to high temperature stress, Hsp90 was significantly increased and then returned to normal levels (Ferreira et al., 2007). In addition, our study has identified one Hsp104 protein belonging to the Hsp100 family which has been shown to be a molecular chaperone in plants (Gurley, 2000), yeast (Glover and Lindquist, 1998), and bacteria (Queitsch et al., 2000). In fact, it has been reported that Hsp104 is the most crucial thermotolerance-related protein of *Saccharomyces cerevisiae*, enhancing survival after exposure to extreme heat or high concentrations of ethanol (Glover and Lindquist, 1998). In our study, similar results were observed. Hsp104 was increased by fourfold under heat stress and then decreased to 1.8-fold after recovery. In mycelium of *P. ostreatus*, Hsp104 is highly expressed and is one of the most important factors for heat resistance. Moreover, Hsp104 provides mycelia with a strong resistance to stress by alleviating the pressure of protein aggregation and promoting degradation of denatured peptide polymers (Bösl et al., 2006). Our study also shows that certain thermo-induced transcription factors show no change in expression under heat stress, but decline in expression levels when returned to normal temperatures. This finding may indicate that these thermo-induced transcription factors may not play a direct role in response to heat stress. In summary, our study suggests that HSPs are key players in *P. ostreatus* heat resistance, and that these components deserve further in-depth study.

The mitogen-activated protein kinases (MAPK) signal pathway is an important signaling system to mediate cell responses (Zhao et al., 2017). The DPs identified in the mycelium under heat stress were found annotating pathway related to MAPK signal pathway, including the cell division control protein 42 homolog, E3 ubiquitin-protein ligase pub1, serine/threonine protein kinase ste20, peroxisomal catalase, MAPK, and MAPK HOG1 involved in maintaining cellular homeostasis. As a signal/pheromone stress regulator protein, MAPK was increased by 2.0-fold under heat stress and then decreased to 1.1-fold after recovery, the expression of this proteins returned to normal level, indicating that MAPK is an important resistant substance in high temperature stress. Moreover, the expression of histidine protein kinase which plays an important role in the hyphal formation and virulence effect decreased to 0.62-fold under heat stress.

### Proteins Metabolism

In our study, it can be seen that many of the proteins involved in metabolism are down-regulated under heat stress suggesting that high temperature affects mycelial metabolism (**Table 5**). However, the expression of phenylalanine ammonia-lyase (PAL) is increased by 5.1-fold under heat stress, and declined 1.5-fold after following recovery, compared to controls. This indicates that PAL may also play a role in the mycelium of *P. ostreatus* under heat stress. PAL catalyzes the first step in the general pathway of biosynthesis of polyphenolic compounds including lignin, cinnamate esters, and flavonoids, and is one of the key enzymes in the metabolism of these compounds. The activity of PAL increases dramatically in response to various stimuli (Jones, 1984). A previous study in Pea Leaf showed that PAL activity has no significant change within 12–14 h, and the activity maximum was at 36–48 h after wounding or jasmonic acid (JA) application. PAL activation induced by wounding or JA lagged far behind the H_2_O_2_ burst. Moreover, the data imply that plasma membrane NADPH oxidase-originated H_2_O_2_ burst is essential for wounding or JA-induced PAL activation (Liu et al., 2008). In our study, similar results were obtained, which might indicate that the accumulation of H_2_O_2_, O_2_^-^, OH^-^ induced by heat stress prompts a significant increase in the expression and synthesis of PAL.

**Table 5:** Variation of proteins involved in metabolism under heat stress and/or subsequent recovery.

Uniprot ID	Mascot score	Fold change		Species	Description
				
		HS/CK1	RC/CK2		
P40235	169.18	1.578	1.316	*Schizosaccharomyces pombe*	Casein kinase I homolog hhp1
O74196	128.48	1.559	1.177	*Colletotrichum gloeosporioides*	Ubiquitin-conjugating enzyme E2–16 kDa
O42939	117.39	0.518	0.975	*Schizosaccharomyces pombe*	Ubiquitin-activating enzyme E1-like
P28345	78.62	2.546	1.018	*Neurospora crassa*	Malate synthase
P52495	70.36	0.576	0.739	*Candida albicans*	Ubiquitin-activating enzyme E1
P43547	63.92	0.583	0.892	*Saccharomyces cerevisiae*	Putative aryl-alcohol dehydrogenase
Q92462	63.24	0.421	0.917	*Schizosaccharomyces pombe*	E3 ubiquitin-protein ligase pub1
P53228	58.98	0.520	0.795	*Saccharomyces cerevisiae*	Transaldolase NQM1
Q12196	55.54	1.592	1.508	*Saccharomyces cerevisiae*	Serine/threonine-protein kinase RIO1
Q03148	51.52	0.492	0.877	*Saccharomyces cerevisiae*	Pyridoxal 5’-phosphate synthase
O13395	50.10	1.593	1.235	*Ustilago maydis*	Chitin synthase 6
B0Y3B5	49.05	0.439	0.623	*Neosartorya fumigata*	E3 ubiquitin ligase complex SCF subunit
O13941	39.02	0.200	0.524	*Schizosaccharomyces pombe*	Uncharacterized beta-glucan synthesis -associated protein
Q6CFX5	37.55	0.599	0.976	*Yarrowia lipolytica*	Serine/threonine-protein phosphatase
P32604	37.08	0.427	0.891	*Saccharomyces cerevisiae*	Fructose-2,6-bisphosphatase
P29465	36.50	0.502	1.225	*Saccharomyces cerevisiae*	Chitin synthase 3
B0CZ32	35.82	0.309	0.702	*Laccaria bicolor*	Methylthioribulose-1-phosphate dehydratase
Q6BWA5	34.53	0.503	0.547	*Debaryomyces hansenii*	Inorganic pyrophosphatase
Q9TEM3	31.60	0.602	0.856	*Emericella nidulans*	2-Methylcitrate synthase, mitochondrial
Q92195	26.99	5.107	1.525	*Agaricus bisporus*	Phenylalanine ammonia-lyase
Q9P3U4	NA^∗^	1.838	1.377	*Schizosaccharomyces pombe*	E3 ubiquitin-protein ligase dbl4


*NA^∗^, the proteins were not quantified under heat stress or subsequent recovery.*

Two additional enzymes involved in cell wall metabolism are chitin synthase (CHS) 3 which is responsible for chitin synthesis and CHS 6 which is involved in its degradation. Chitin production involves a dynamic balance between CHS and the chitin degradation enzyme, chitinase (Rogg et al., 2012). Interestingly, the expression of these two proteins are opposite in response to thermal stress. CHS 3 is reduced by 0.5-fold under heat stress, and CHS 6 is increased 1.6-fold, indicating that CHS 6 plays a dominant role in cell wall integrity and stress. Another protein involved in cell wall synthesis and degradation is the uncharacterized beta-glucan synthesis-associated protein. Its expression declined fivefold under heat stress, indicating that the cell wall of hyphae may have suffered serious damage under heat stress. In addition, triose phosphate isomerase, glutamate synthetase, and affinity phosphate permease, and inorganic pyrophosphatase are down-regulated. Again, this supports the hypothesis that high temperature stress affects hyphal biosynthesis and metabolism.

## Conclusion

An iTRAQ-based proteomic technique was employed to compare the abundance of proteins in heat stress and/or subsequent recovery of *P. ostreatus* mycelium culture for 48 h. Two hundred and four DEPs were identified. These DEPs are mainly involved in the biological processes of cellular, metabolic, multi-organism, reproductive, and developmental processes; biological regulation; localization; nitrogen utilization; cellular component organization or biogenesis; reproduction; response to stimulus; signaling and growth biological processes. The diverse array of proteins affected by heat stress conditions and subsequent recovery indicates that there is a remarkable flexibility in mycelium metabolism, which may contribute to its survival in heat stress. The morphological combined with physiological analysis that the iTRAQ-based proteomic technique is sufficiently reliable for the identification and quantification of a large number of mycelium. qRT-PCR results suggest that the expression of some proteins (e.g., malate synthase) can be regulated by post-transcriptional modifications. With iTRAQ-based proteomic technique, many new heat-responsive proteins, such as PAL, LDS, and MAPK, were identified from *P. ostreatus* mycelium. These novel proteins provide a good starting point for further research into their functions using genetic or other approaches. These findings significantly improve the understanding of the molecular mechanisms involved in the tolerance of fungi to heat stress.

## Author Contributions

YZ, MZ, and JQ conceived, designed, and performed the experiments, analyzed the data, and wrote and revised the manuscript. JZ conceived and designed the experiments.

## Conflict of Interest Statement

The authors declare that the research was conducted in the absence of any commercial or financial relationships that could be construed as a potential conflict of interest.

## Footnotes

1 https://david.ncifcrf.gov/

2 http://www.genome.jp/kegg/mapper.html

3 https://string-db.org/

4 https://www.uniprot.org/

5 http://www.ncbi.nlm.nih.gov/BLAST/

6 http://www.uniprot.org/uniprot/Q6BZH1-Function
